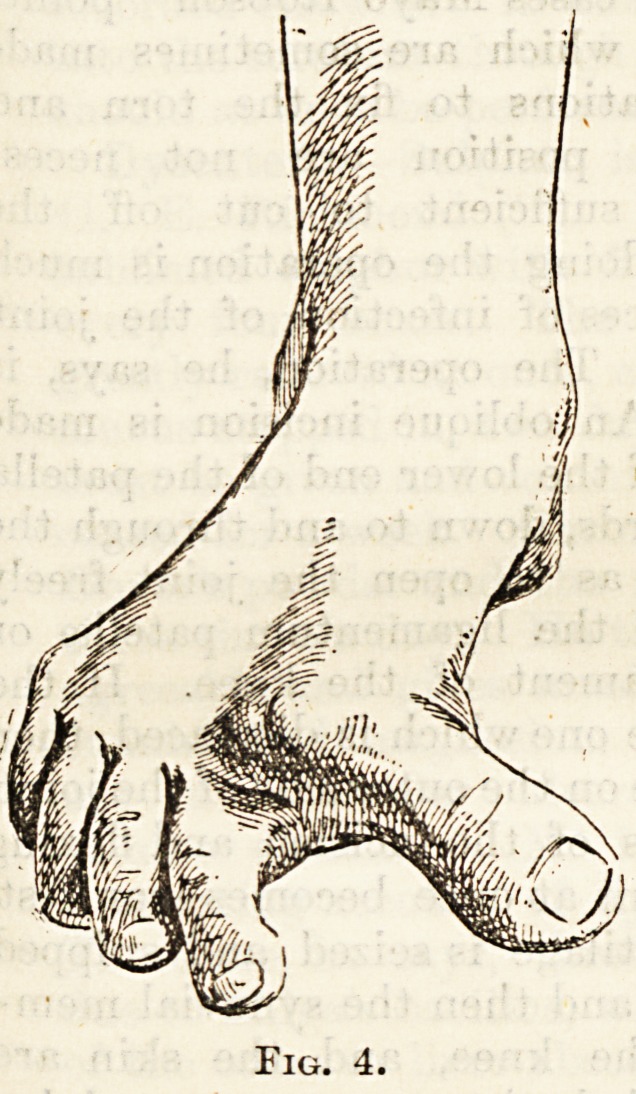# Hallux Valgus, or Bunion

**Published:** 1902-04-19

**Authors:** John Poland

**Affiliations:** Surgeon to the City Orthopædic Hospital, Senior Surgeon to the Miller Hospital.


### Hospital Clinics and Medical Progress.

### HALLUX VALGUS, OR BUNION.

By John Poland, F.R.C.S., Surgeon to the City Orthopædic Hospital, Senior Surgeon to the Miller Hospital.

The occurrence of hallux valgus, or outward displacement of the great toe, from its mildest to its most severe form, is so common in modern times that anything that will assist us in its true etiology and pathology is of interest. This is the most common of all deformities of the foot. The majority of cases are, as stated in text-books, due to badly fitting and improperly shaped boots, pointed-toe boots, etc. With these sources, however, there is often combined an hereditary tendency to rheumatism, gout, and other arthritic diseases. I will not allude in this paper to hallux valgus as seen in adults, and due to ill-fitting boots or shoes. In the type with which I am here concerned the deformity is not essentially due to that cause, but is a distinct class, to which proper attention has never been given, and of which I have seen numerous instances during the past few years—namely, bunions of congenital origin. They are of the greatest interest in a pathological sense, and have all occurred in patients below 20 years of age. In some of them the deformity existed in a marked degree ; yet in works on surgical diseases in children no mention is made of the possible occurrence of such a condition or of the possibility of correcting it by timely employment of properly constructed boots and shoes. The displacement outwards of the first phalanx of the toe with the sesamoid bones and extensor longus hallucis is the same as in the adult, and later on a bunion may be developed over the inner side of the head of the metatarsal bone. In some cases it can only be said that the condition is hereditary ; that is, the deformity only becomes marked after some exciting cause, such as improper boots. It is possible that this hereditary tendency may be due to contraction of the external lateral ligament of the joint and analogous to the contracted ligament seen in hereditary hammer-toes. Sometimes the deformity is confined to one foot, the opposite one being quite normal, as in the first case mentioned below (fig. 1). In other cases it is bilateral, as in figure 2. According to Meyer's line, in the natural foot of children the long axis of the great toe continued backwards passes through the centre of the heel. Any outward deviation of the great toe from this line constitutes hallux valgus. I have not seen the congenital form of the deformity associated with hammer-toe, which is well known to be often hereditary, sometimes congenital. Hammer-toe in older persons is frequently the result of hallux valgus, and in these instances is entirely secondary to the displacement outwards of the great toe. But in the form under consideration I have not met with any tendency to flat foot similar to that so frequent in older persons.

In the following case, in a boy aged fourteen, the valgus condition was unilateral. There was great displacement of the great toe outwards, so that it made a very obtuse angle with the hinder part of the foot. The head of the first metatarsal bone was very prominent, but no bunion or bursa existed over it such as is so commonly seen in the ordinary form of hallux valgus in adults (see fig. 1). There was no paralytic condition nor spastic contraction, and the boy had never worn tight or ill-shaped boots ; they had always been large and well made. The usual causes of this disease, therefore, were not present in this case. The deformity had become very noticeable during the last three or four years.

Fig. 1.

Believing that it might be due to some slight injury to the bone or to the epiphysial line of cartilage sometimes met with at this end of the bone—of which there was, however, no history—I removed the head of the metatarsal bone, with a good result except that later on the extensor tendon had to be divided. After examining microscopically the piece of bone I could make out no trace of injury, and the lines of stress of the cancellous tissue were normal. The structure of the bone and articular cartilage were quite normal, with the exception that the latter was degenerated on the inner side. As usual, the inner side of the end of the bone was prominent, but without any bursal formation over it. The facets for the sesamoid bone were displaced to the outer side and very close together, the inner facet being in contact with the outer side of the internal condyloid portion of the articular cartilage. Even after removal it was difficult to say what was the precise pathological condition. On making a section of the head of the bone, and comparing it with other normal bones, no definite conclusion could be come to. Sometimes there is a mushroom-shaped epiphysis at the head of the metatarsal bone, but there was no trace of that here, so that the deformity could not have been the result of injury and irregular growth of an epiphysial disc. It was clearly the result of some congenital defect. The case did exceedingly well, and the patient is now walking about in a perfect condition.

In the case just mentioned there was no mechanical cause, such as pressure by boots, and the same held good in the case of a girl, aged 17, from whom also I removed the head of the metatarsal bone. In both these specimens it is interesting to note that there were two distinct facets for the sesamoid bones to glide over, and there were the usual displacements towards the outer side and partial dislocation at the metatarso-phalangeal joint. In the case of the girl the deformity was bilateral ; the head of the metatarsal bone was removed on one side, and on the other the external lateral ligament and the adductor obliquus hallucis tendon were divided, as the condition was more intense on the one side than the other. None of the usual causes for the deformity could be established ; some pain in walking had been experienced since infancy, and that pain had been increased during the last few years. The deformity was not noticed at birth, and it was only when the pain developed that attention was drawn to it. The result of the operation was excellent.

What the actual pathological cause in these cases was it is impossible at present to say, but that they do form a distinct class cannot be doubted, and I think they should be so considered in works on orthopædic surgery.

The accompanying skiagram (fig. 2) shows the hallux valgus on the right side in the case of the girl corrected after the syndesmotomy and tenotomy, and on the left side the appearance after excision of the head of the metatarsal bone with absence of the previous existing deformity. In this foot the skiagram displayed a fusiform enlargement towards the anterior end of the shaft of the second metatarsal bone, the nature of which was not determined; but there were no symptoms connected with it.

Fig. 2.

In the milder forms of deformity division of the external lateral, and outer fibres of the capsular ligament of the metatarsal phalangeal joint and the use of a bunion spring to the inner side of the toe and foot may be sufficient to effect a cure. Sometimes it may be advisable to divide the adductor obliquus hallucis and adductor transversus hallucis tendons, as in the case described above. It may even be necessary to divide the extensor hallucis longus, which is displaced in extreme deformities to the outer side of the metatarso-phalangeal joint and aggravates the valgus condition. A toe-post with properly constructed boots and digital stockings are useful in the more advanced deformities. When the deformity has reached a severe degree, excision of the head of the metatarsal bone is the only operation that is satisfactory. The operation is at once simple, and in the five instances in which I have performed it for this variety of hallux valgus it has proved of eminent benefit, having been most effectual and wholly successful. The results are more satisfactory, indeed, than after excision of a wedge of the metatarsal bone or partial excision of the head of the bone.

The operation is thus performed, the foot is carefully cleansed, the parts rendered thoroughly aseptic in the usual way by biniodide of mercury, etc. A longitudinal incision, two inches long, is made on the upper and inner aspect of the metatarso-phalangeal joint down to the bone. After the joint has been freely opened, the head of the metatarsal bone is easily exposed and freed by the point of the scalpel. Bone forceps are used for dividing the bone. The base of the phalanx is left. The wound is accurately closed by two or three silkworm gut sutures and carefully dressed with iodoform gauze and wool. The toe is kept in a straight line by means of a malleable tin splint applied to the inner side of the toe and foot. The dressings are kept on for two or three weeks, sometimes even longer. The subsequent use of properly made and well-fitting boots and shoes is of course essential.

Fig. 3

Children with the hereditary tendency I have indicated should, during the early period of childhood at any rate, both summer and winter, wear Roman sandals similar to those made by Messrs. Hall and Son, Limited, of Bishopsgate Street, City. They are made of stout leather, the exact shape of the child's foot (see fig. 3), with a supporting leather thong between the great and second toe. This is an excellent pattern and one to be recommended. If boots are to be worn, they should follow the natural shape of the foot; that is, straight on the inner and curved on the outer side. Ill-shaped boots and shoes made with the inner and outer lines of the sole converging to a point between the second and third toe are responsible for the milder form of hallux valgus so frequently seen in young children. Indeed, these mild forms of hallux valgus, the consequences of wearing the median-pointed boots, are believed by some surgeons to be almost universal among children of the middle classes, especially among girls. This acquired form of valgus will not be discussed here.

In conclusion I will mention, by way of contrast, the rare affection of hullux varus or pigeon-toe, the exact reverse of hallux valgus. The great toe is displaced inwards from the other toes, being in the abducted position from the middle line of the foot. When the displacement is great, division of the internal lateral ligament of the joint and the abductor hallucis tendon and replacement of the toe in its normal position will be found necessary, as in the following case, where it was most succesful. Fig. 4 is taken from the cast of the foot of the case.

Fig. 4.

The boy, aged 14, was said to have had club foot from birth, but no treatment had been given to it. The displacement of the right great toe had only made its appearance gradually during the last two years. There was a slightly wasted appearance of the limb, and an atrophied and contracted condition of the abductor hallucis. These symptoms, together with a slight tendency to the equino-varus position of the foot, suggested that this deformity was a paralytic one. After division of the abductor hallucis and plantar fascia on the inner side of the foot, and later on of the glenoid and internal lateral ligaments of the metatarso-phalangeal joint and use of a metal splint, the normal position of the toe was perfectly restored.

A unique case, as far as I am aware, of congenital hallux varus in both feet came under my care a short time ago in a child aged two years. The condition as displayed by the skiagram was a very remarkable one. The second phalanx of the great toe was completely displaced to the inner side of the first phalanx and was in all probability a congenital dislocation of the joint analogous to what is seen in congenital dislocation of the hip. That there were very great developmental errors in the feet was also shown in a very remarkable way, in that all the epiphyses of the bones of the feet were different from normal. On looking at the first phalanx it was seen that the nucleus of the proximal end of all the phalanges had already appeared but the nucleus for the heads of the metatarsal bones had not done so, although the first metatarsal bone had here a nucleus like the other phalanges which should not normally appear until the third and fourth year. Whether it was a ligamentous contraction giving rise to the deformity of the toes could not be ascertained; indeed I have not seen any skiagram of this condition published, and no opportunity of dissecting a specimen of this kind has ever occurred. It was proposed to operate and replace the toes in position.

## Figures and Tables

**Fig. 1. f1:**
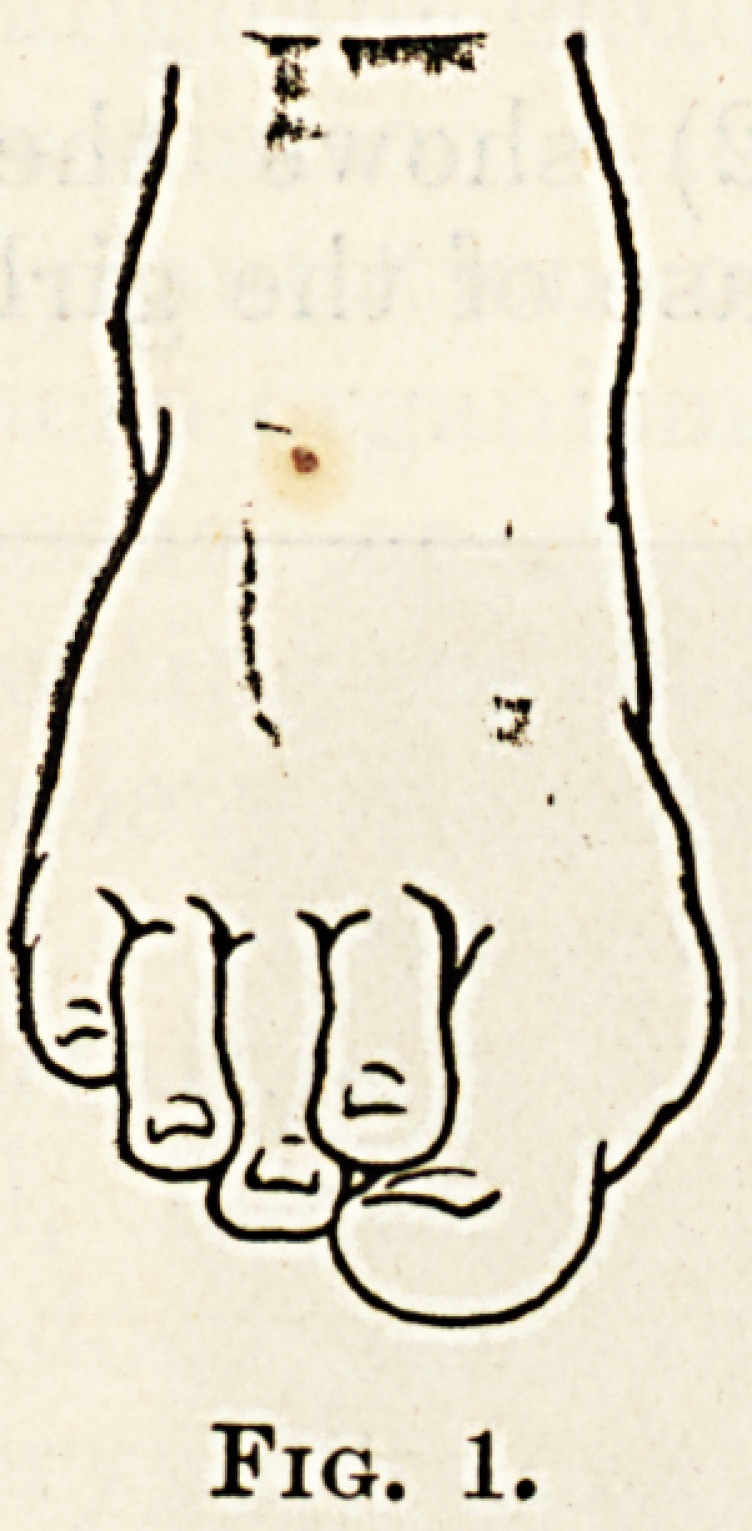


**Fig. 2. f2:**
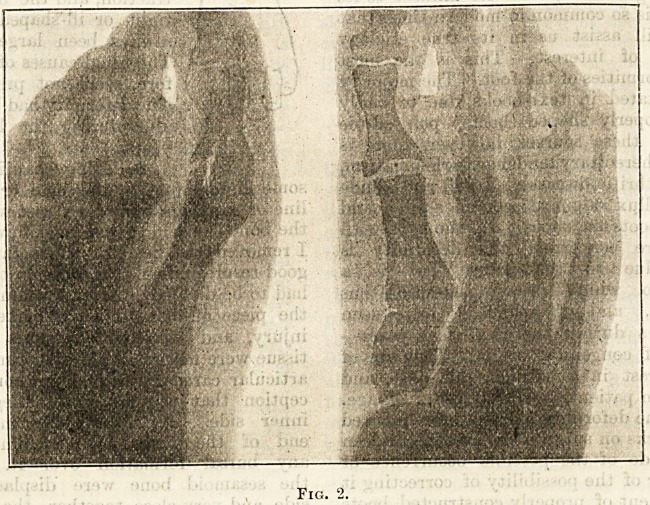


**Fig. 3. f3:**
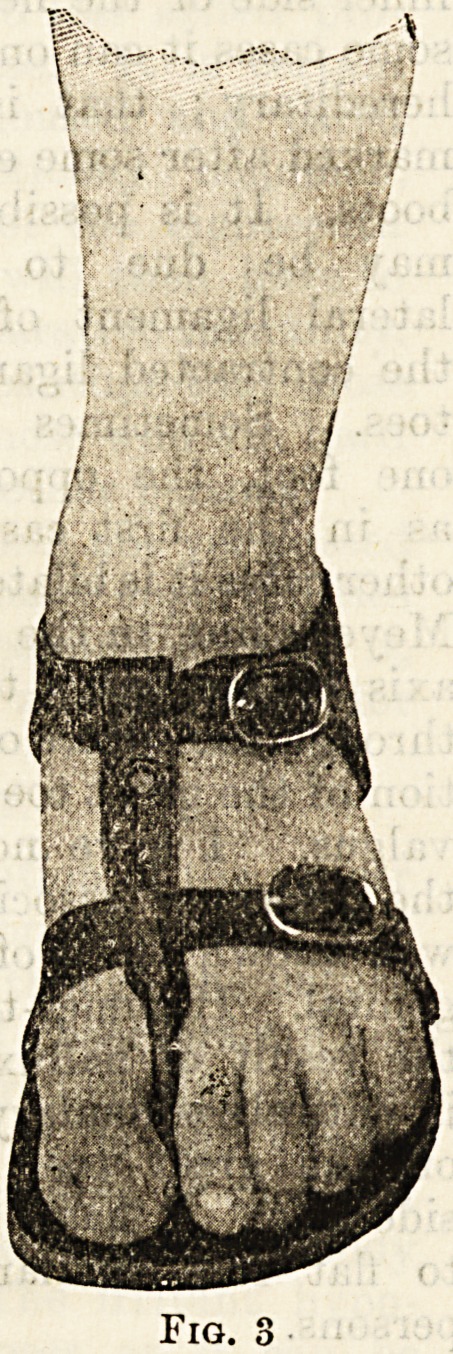


**Fig. 4. f4:**